# Domestic horses (*Equus caballus*) prefer to approach humans displaying a submissive body posture rather than a dominant body posture

**DOI:** 10.1007/s10071-017-1140-4

**Published:** 2017-10-13

**Authors:** Amy Victoria Smith, Clara Wilson, Karen McComb, Leanne Proops

**Affiliations:** 1 Mammal Vocal Communication and Cognition Research Group, School of Psychology, University of Sussex, Brighton, BN1 9QH UK; 2 Centre for Comparative and Evolutionary Psychology, Department of Psychology, University of Portsmouth, Portsmouth, PO1 2DY UK

**Keywords:** Body posture, Interspecific communication, Emotion recognition, Dominance and submissiveness, Horse–human relationship

## Abstract

Signals of dominance and submissiveness are central to conspecific communication in many species. For domestic animals, sensitivities to these signals in humans may also be beneficial. We presented domestic horses with a free choice between two unfamiliar humans, one adopting a submissive and the other a dominant body posture, with vocal and facial cues absent. Horses had previously been given food rewards by both human demonstrators, adopting neutral postures, to encourage approach behaviour. Across four counterbalanced test trials, horses showed a significant preference for approaching the submissive posture in both the first trial and across subsequent trials, and no individual subject showed an overall preference for dominant postures. There was no significant difference in latency to approach the two postures. This study provides novel evidence that domestic horses may spontaneously discriminate between, and attribute communicative significance to, human body postures of dominance; and further, that familiarity with the signaller is not a requirement for this response. These findings raise interesting questions about the plasticity of social signal perception across the species barrier.

**Electronic supplementary material:**

The online version of this article (doi:10.1007/s10071-017-1140-4) contains supplementary material, which is available to authorized users.

## Introduction

In species with dominance hierarchies, the effective communication of rank and ability are crucial for maintaining social relationships and managing access to resources (Kaufmann 1983). Displays of dominance and submissiveness are often linked to affect, e.g. aggression in dominant displays and fear in submissive displays (Drews 1993). Dominance-related communicative body postures are widespread and may be evolutionarily conserved due to similarities in form across species: dominant postures tend to involve an inflated body size whilst submissive postures involve making oneself appear smaller and less threatening (Darwin 1872; Miller 1995).

Domestic animals are likely to benefit from recognising communicative human signals such as facial expressions of emotion (e.g. Racca et al. 2012; Smith et al. 2016), though little empirical research has directly investigated animals’ abilities to interpret human postural cues. There is, however, some evidence that piglets preferentially approach crouching versus erect humans, suggesting an avoidance of larger, potentially more threatening, body postures (Miura et al. 1996), and that dogs respond to humans adopting typical ‘play’ postures (bowing and lunging) by increasing their own play behaviour (Rooney et al. 2001). These findings suggest that human body posture cues can be influential signalling components in human–animal interactions.

Horses are a highly social, herd-living species that maintain strict dominance hierarchies through the use of visual cues such as body posture (Waring 2003). Although some equine training techniques utilise larger and smaller human postures as negative and positive training cues, respectively, evidence for horses’ discrimination of these postures is lacking (Henshall and McGreevy 2014). Previous research has shown no difference in the approach rates of horses to stationary humans adopting aggressive versus submissive postures (Seaman et al. 2002), or in the flight distance of feral ponies when approached by tense versus relaxed humans (Birke et al. 2011). These results may, however, be due in part to the paradigms offering no reward incentives to encourage horses to interact with the humans.

This study explores whether domestic horses discriminate between human body postures of dominance and submissiveness after being trained to approach the human demonstrators adopting a neutral posture. A two-choice paradigm was used where one demonstrator adopted a dominant and the other a submissive posture, and horses were free to approach either demonstrator over four trials. Vocal and facial cues were absent, so we could investigate the specific importance of bodily cues. Approach rates and latencies to approach dominant and submissive postures were measured. Horses’ responses may shed light on the social significance of dominant body signals and the plasticity of posture cue recognition across species.

## Methods

### Study animals

Forty-five domestic horses were recruited from three equestrian centres in Suffolk and East Sussex, UK. Six horses failed to reach criterion in the warm-up phase and nine developed a side bias (choosing the same side in all four trials), and therefore, 30 subjects were included in the final analysis (22 geldings, 8 mares; ages 7–26, *M* = 18.2, SD = 5.43). One horse failed to complete all four test trials and was excluded from preference analyses (successful trials, *N* = 1). Owner records ensured that all horses were comfortable being handled by unfamiliar humans and had no known eyesight problems. No horses were food deprived during the study.

### Human demonstrators

**Fig. 1:**
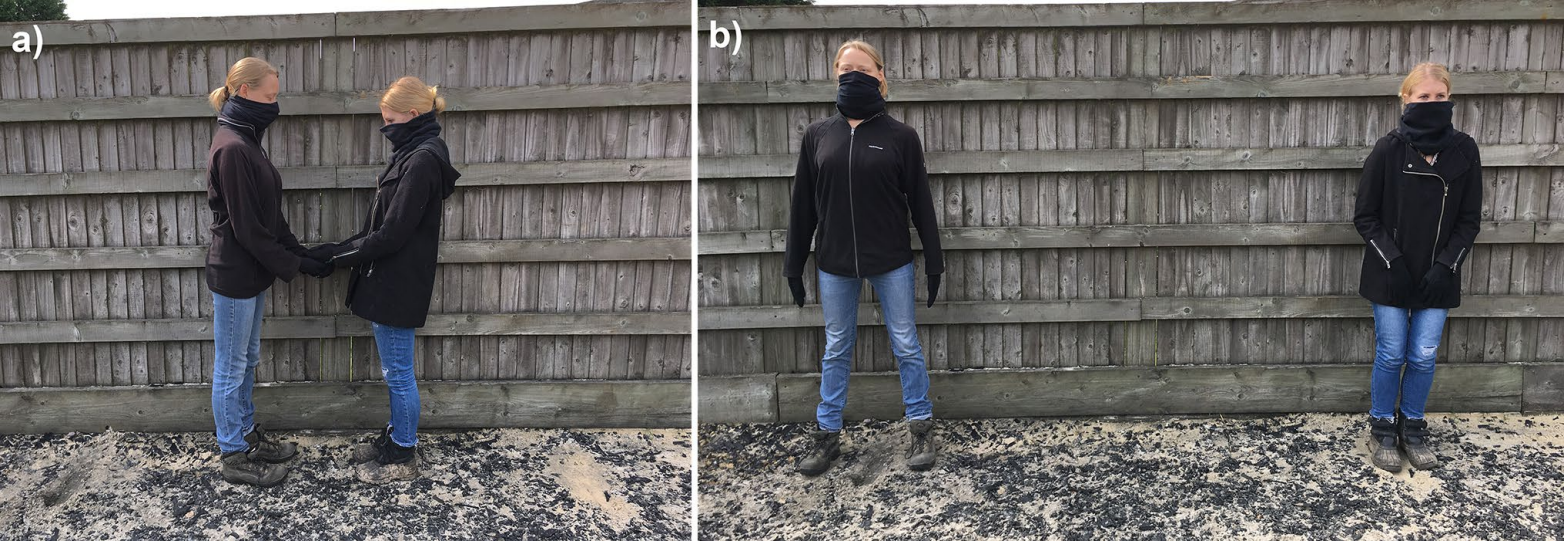
Example of demonstrators’ positions during **a** a warm-up trial and **b** a test trial (dominant on the left; submissive on the right)

Ten adult females acted as demonstrators. All wore dark jumpers/jackets, black gloves, jeans or trousers, and a dark neck warmer covering the face to eye level to minimise facial expression cues. Demonstrators were approximately matched by overall build. To reduce potential behavioural cueing, demonstrators were told that there is conflicting evidence for horses preferring both dominant and submissive postures. Two demonstrators were aware of the responses given by previous horses, and this did not significantly influence horses’ responses (see the “Behavioural analysis” section below). During test trials, demonstrators looked directly forwards without making eye contact with the horse. Detailed posture instructions are described in Table 1. Practice sessions ensured consistency across individuals and trials. Only four horses approached the same demonstrator in all four test trials, with no individual demonstrator being preferred by more than one horse. Due to the large number of demonstrators (*N* = 10) relative to the number of subjects (*N* = 30), statistical analysis of demonstrator preference was not included in the paper. Examples of the postures used during Phase 1 and Phase 2 are shown in Fig. 1a, b.

**Table 1:** Definitions of test trial postures for demonstrators

Posture	Description
Dominant	Standing tall^a,b^; feet hip-width apart^c^; squared shoulders^a^; chest puffed out^b,d^; hands to the side^c^; an ‘open’ body posture^c^
Submissive	Slouching^d,e^; feet together^c^; hunched shoulders^a,d^; relaxed knees^c^; hands to the front^c,e^; a ‘closed’ body posture^c^

^a^Seaman et al. (2002); ^b^Argyle (1988); ^c^Cashdan (1998); ^d^Kudoh and Matsumoto (1985); ^e^Tiedens and Fragale (2003)

### Procedure

Horses were tested individually in familiar riding school arenas. The same handler was used for each test. Horses’ responses were recorded on two wide-angled Panasonic HD-V720 cameras located directly behind and to the left of the experimental area. The experiment consisted of an initial warm-up phase (Phase 1) followed by four test trials (Phase 2).

#### Phase 1: Warm-up trials

**Fig. 2:**
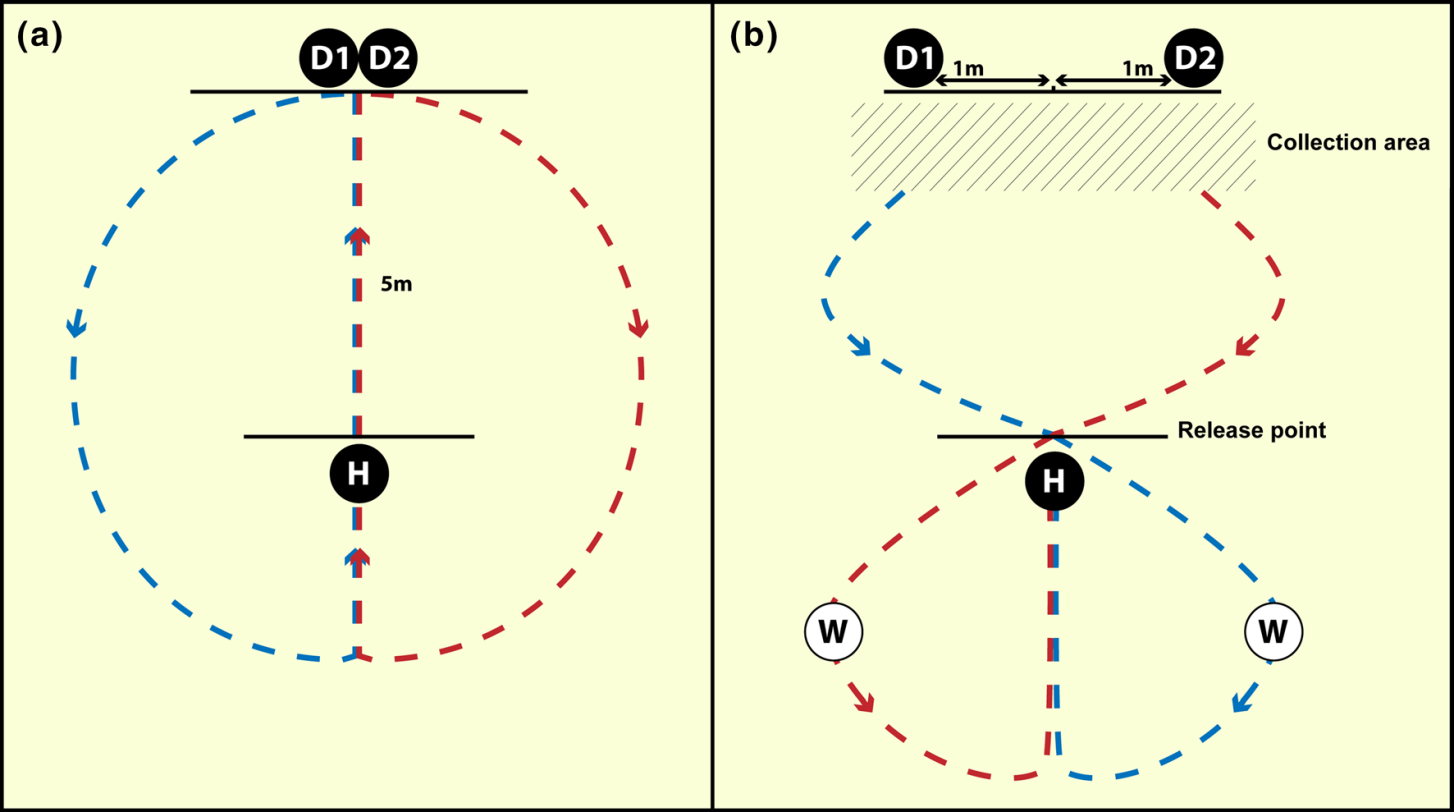
Experimental set-up of **a** warm-up trials and **b** test trials. D1 and D2 = demonstrators; H = horse’s starting point; W = wait points; red and blue lines = paths alternated between trials to avoid side biases

The purpose of warm-up trials was to encourage horses to approach the human demonstrators. Figure 2a provides a schematic of the warm-up trials set-up. This phase was considered successful when the horse reliably approached the demonstrators from a 5-m release point twice (trials required: *M* = 6.8, SD = 1.65). Horses failing to reach criterion within 10 trials did not progress to test trials (*N* = 6). As Fig. 1a shows, in each warm-up trial, two demonstrators stood facing each other with both arms bent at the elbow and hands overlapping, together holding one piece of carrot. The horse was led along the 5-m centre line to receive the carrot and then was led away in the opposite direction from the previous trial to prevent side biases developing. During warm-up trials demonstrators adopted a neutral posture with feet slightly apart and head pointed slightly down.

#### Phase 2: Test trials

Following warm-up trials, horses were led to the wait point and held facing away from the demonstrators. Figure 2b provides a schematic of the test trial set-up. Demonstrators then stood 2 m apart, one displaying the submissive and the other the dominant posture. Horses were led to the 5-m point and released, allowing them to approach the demonstrator of their choice. Approaches were defined as the horse’s nose reaching within 50 cm of one demonstrator. Immediately after a choice was made, the horse was collected from the collection area and led away in the opposite direction to the previous trial. No food rewards were given during test trials; however, between each test trial, immediately after a successful choice, horses were given a reinforcement trial to maintain their approach behaviour. Reinforcement trials followed the same procedure as warm-up trials.

Once the subject had received the reinforcement they were led away in the opposite direction to the previous trial in a figure-of-eight shape and were held at the wait point for 30 s before starting the next test trial, which has been shown to reduce side biases (Proops and McComb 2010). If a horse failed to approach, the test trial was repeated. Where subjects lost motivation to approach, up to two additional reinforcement trials were permitted (*N* = 9). If subjects did not regain motivation, the test was discontinued, and only the successful trials were recorded for that subject (*N* = 1). Each test was counterbalanced such that for every horse, over a set of four trials, each demonstrator served as submissive twice and dominant twice, and each demonstrator displaying each posture was presented on the left twice and the right twice. This produced 24 possible permutations in the order of presentations. These permutations were assigned randomly between horses and counterbalanced such that all permutations were used at least once.

### Behavioural analysis

Behavioural measures were the horses’ choice (dominant or submissive) and their mean latency to approach (time between stepping over the release point line and approaching a demonstrator). In three trials, the latency could not be computed due to technical issues and so data was entered as missing. Ten videos (33.3%) were double-coded for reliability showing 100% agreement on choice of posture and good reliability for latency to approach (single-measures absolute agreement ICC of 0.83). Two demonstrators were not blind to the horses’ responses in previous trials but this did not significantly affect the horses’ probability of choosing dominant or submissive postures, *χ* ^2^(4) = 0.52, *P* = 0.97.

## Results

### Posture choice

**Fig. 3:**
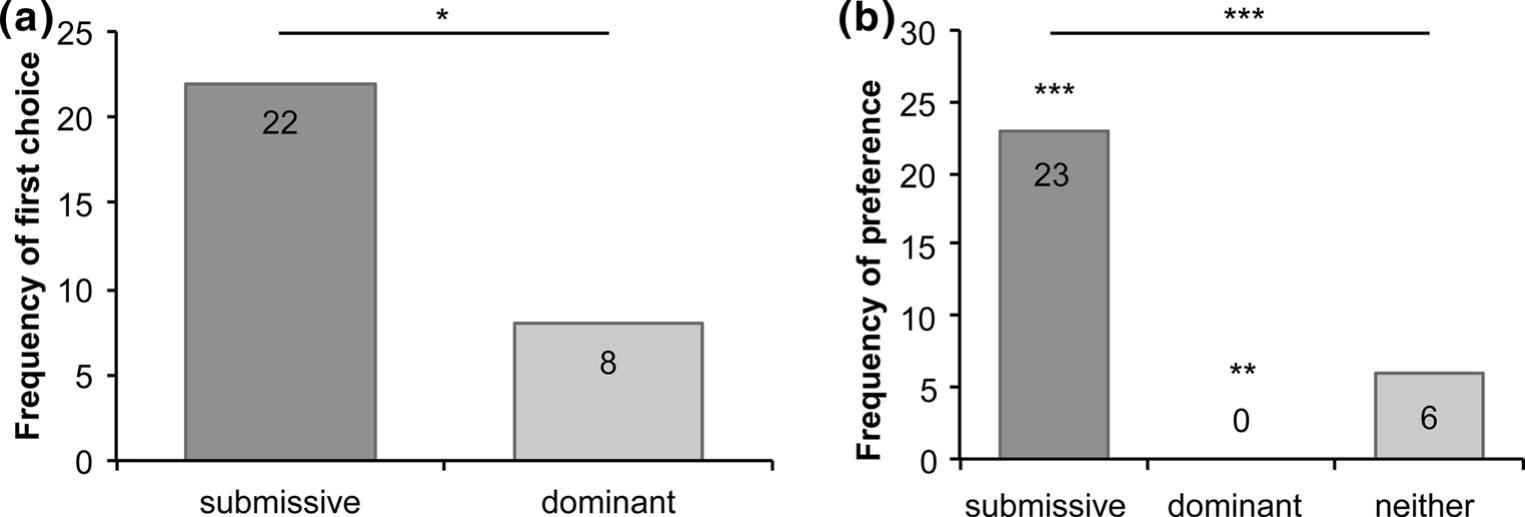
**a** Frequencies of first approach by posture type; **b** frequencies of preference scores by posture type, *P < 0.05, **P < 0.01, ***P < 0.001

Across all test trials horses performed 90 approaches to submissive and 27 approaches to dominant postures. Figure 3a shows that horses were significantly more likely to approach submissive over dominant postures as their first posture choice, *N* = 30, *K* = 22, *P* = 0.016 (binomial probability).

Preference was defined as a horse choosing one posture in more than half of the test trials (i.e. in 3 or 4 out of 4 trials) or no preference (2 choices for submissive; 2 choices for dominant). A Chi-square goodness-of-fit test showed that the distribution of horses across preference scores (submissive, *N* = 23; dominant, *N* = 0; no preference, *N* = 6) was not consistent with the null hypothesis, *χ* ^2^(2) = 32.68, *P* < 0.001, as shown in Fig. 3b. To investigate the contributions of individual cells to the Chi-square results, standardized residuals were inspected, where values outside ± 1.96 indicate significance at alpha 0.05 level; ± 2.58 at the 0.01 level; and ± 3.29 at the 0.001 level (Field 2009, p. 699). Residuals indicated that horses chose submissive postures significantly more than expected by chance, *z* = 4.63, *P* < 0.001, and dominant significantly less than expected, *z* = − 3.01, *P* < 0.01, with no significant difference for no preference, *z* = − 1.48, *P* > 0.05 (see Online Resource for table of results).

An ordinal regression showed no significant influence of age or sex on the proportion of posture choice (choosing submissive in 0, 25, 50, 75, or 100% of trials), *χ* ^2^(2) = 0.42, *P* = 0.81 (sex, Wald *χ* ^2^(1) = 0.006, *P* = 0.94; age, Wald *χ* ^2^(1) = 0.39, *P* = 0.53).

### Latency to approach

In the majority of trials (89.5%), horses approached within 10 s (sample: *M* = 6.29, SD = 7.32: range 2.04–50.09 s). Trials with latencies > 10 s were excluded as outliers (*N* = 12, with 10 submissive and 2 dominant; new sample: *M* = 4.21, SD = 1.08; range 2.04–8.89 s), and latencies were normalised by log transformation.

A linear mixed model (trial as a repeated measure, subject as a random effect) investigated potential differences in horses’ latencies to approach submissive (*M* = 4.20, SD = 1.07) and dominant (*M* = 4.25, SD = 1.14) postures. Model fit was determined using Akaike’s information criteria for small samples (AIC_c_) where smaller scores indicate a better model. This showed no significant effects of age, sex, posture choice, or whether the horse approached both versus only approached one of the postures (see Online Resource for a table of AIC_c_ and ΔAIC_c_ scores), with the intercept-only model being the best fit.

## Discussion

Horses significantly preferred to approach a submissive versus dominant posture, with no individual showing an overall preference for approaching the demonstrator adopting a dominant posture. These results demonstrate horses’ ability to spontaneously discriminate between human body postures without explicit training and towards unfamiliar individuals. However, no difference was observed in approach latency.

Horses’ preference for submissive postures could be explained by either an avoidance of the dominant, as larger postures are typically used in threatening contexts (Kaufmann 1983), or an attraction to the submissive as a signal of appeasement or compliance (Allan and Gilbert 1997). Horses typically avoid dominant conspecifics; however, they also follow dominant horses towards food sources (Andrieu et al. 2016), and so the adaptive significance of approaching or avoiding dominant individuals is likely to be complex. This may account for the lack of difference in approach latency to dominant human postures. To determine whether submissive postures are inherently attractive, further research could include a ‘neutral’ posture to assess whether horses prefer submissive over neutral postures.

Importantly, only two postures were used in this initial investigation, and therefore the results cannot be generalised to all postures of dominance and submissiveness. Here, some variation was introduced through the use of ten different demonstrators; however, future studies should use several different dominant and submissive postures to further increase the generalizability of the current results. It is also possible that individual differences between demonstrators in odour and clothing may have influenced their attractiveness to horses. However, ten different demonstrators were used to introduce variation and each demonstrator served as dominant and submissive in two out of four trials, thus an experimenter bias could not produce a corresponding posture preference.

These results raise interesting questions about possible universality and flexibility of dominance signalling across species. Such findings serve to enhance our understanding of interspecific communication and are relevant for informing horse handlers and trainers about the ways horses perceive our communicative signals.

## Electronic supplementary material

Below is the link to the electronic supplementary material.

Supplementary material 1 (PDF 108 kb)
