# Supplementary material for: Domestic horses (Equus caballus) prefer to approach humans displaying a submissive body posture rather than a dominant body posture
Source: Anim Cogn. 2017 Oct 13;21(2):307–12. doi: 10.1007/s10071-017-1140-4 (PMC5818628; doi:10.1007/s10071-017-1140-4)
Supplement: Supplementary file 1 — Supplementary material 1 (PDF 108 kb) [file 10071_2017_1140_MOESM1_ESM.pdf]

Electronic Supplementary Material

Article: Domestic horses (*Equus caballus*) prefer to approach humans displaying a submissive body posture rather than a dominant body posture. Smith AV, Wilson C, McComb K, & Proops L, *Animal Cognition.*

Corresponding authors: Amy Victoria Smith, Uni. Sussex: a.v.smith@sussex.ac.uk; Leanne Proops, Uni. Portsmouth: leanne.proops@port.ac.uk

Table 1. Frequencies and standardized residuals assessing the deviation from chance for frequencies of preference scores

Preference over 4 trials	Observed frequency (*N*)	Expected proportion	Standardized residuals	*P*
**Submissive**	23	0.3125	+4.63	>0.001
**Dominant**	0	0.3125	-3.01	>0.01
**No preference**	6	0.375	-1.48	<0.05

Table 2. Linear mixed model AIC_c and AIC_c change (ΔAIC_c) scores for horses' latencies to approach demonstrators

A priori **hypothesised models**	AIC_c	ΔAIC_c
**Global model: posture choice + sex + age + approach both postures (yes/no) + posture choice*sex + posture choice*age**	-225.238	
**posture choice + sex + age + approach both postures (yes/no) + posture choice*sex**	-227.539	-2.301
**sex + age + approach both postures (yes/no) + posture choice*sex**	-227.539	0
**age + approach both postures (yes/no) +**	-227.539	0

**posture choice*sex**		
**age + approach both postures (yes/no)**	-231.511	-3.972
**age**	-233.149	-1.638
**intercept only**	-233.143	0.006
